# Design of a Hollow Fiber Supported Liquid Membrane System for Zn Speciation in Natural Waters

**DOI:** 10.3390/membranes8040088

**Published:** 2018-09-27

**Authors:** Clàudia Fontàs, Enriqueta Anticó, Victòria Salvadó

**Affiliations:** Department of Chemistry, University of Girona, C/Maria Aurèlia Capmany 69, 17003 Girona, Spain; claudia.fontas@udg.edu (C.F.); enriqueta.antico@udg.edu (E.A.)

**Keywords:** hollow fiber, supported liquid membrane, D2EHPA, zinc, speciation, natural waters

## Abstract

A supported liquid membrane-hollow fiber system (HFSLM) has been developed to determine zinc speciation in aquatic environments. The liquid membrane consisted of an organic solution of bis-(2-ethylhexyl)phosphoric acid (D2EHPA) impregnated in the microporous of a polypropylene hollow fiber. The membrane contacted both the donor solution, that contained the metal and the stripping solution, placed in the lumen of the hollow fiber, where the metal was preconcentrated. Different parameters affecting the Zn^2+^ transport efficiency have been evaluated such as the composition of both the donor and stripping solutions as well as the membrane phase. Extraction and transport efficiencies of free Zn(II) higher than 90% were obtained with a liquid membrane consisting of a 0.1 M D2EHPA solution in dodecane and a 0.1 M HNO_3_ solution as the stripping phase. The developed HFSLM was used to study the effect of different ligands (EDTA and citric acid) in the donor phase of Zn(II) transport and to investigate the selectivity of the membrane towards Zn when other metals were also present. Finally, the HFSLM system was successfully applied to estimate the free Zn(II) concentrations in three water samples from a mining area. Moreover, the HFSLM system facilitates the analytical determination of trace Zn(II) levels allowing the achievement of enrichment factors of around 700 in the stripping phase.

## 1. Introduction

The total concentration of metals present in the aquatic environment does not provide information regarding their distribution, mobility, biological availability and toxicity. The different species in which metal ions may be present in the aquatic environment lead to different types of interactions with biological membranes depending on their bioavailability [1]. The metal bioavailability depends on their chemical speciation and, in general, it is considered that the most bioavailable metallic species are dissolved and that the amount of metal that can be bio-assimilated by a living organism is proportional to the fraction of free metal or labile metal species present in the media [2]. Moreover, metal bioavailability also depends on physicochemical factors such as temperature, salinity, the presence or absence of complexing agents, the presence or absence of other metals and the characteristics and state of the organism [3,4]. Among the different metal species that are present at trace concentration levels in natural waters, some of them are considered toxic (e.g., Cd, Hg and Pb species) while others are nutrients for plants and animals (e.g., Cu, Fe, Mn, Zn species) although they can be toxic at elevated concentrations. Zn is an essential trace metal micronutrient needed by plants to perform various biochemical reactions and physiological functions [5]. However, an excess of Zn can produce oxidative stress in plants and toxic effects in microalgae and other aquatic organisms [6].

Different chemical-based techniques, such as the Donnan membrane technique (DMT), diffusion gradients in thin films (DGT), and permeation liquid membranes (PLM), have been developed to assess the bioavailable metal fraction in natural systems [7]. PLM consists of an organic solution immobilized onto a suitable inert microporous support interposed between two different aqueous solutions. This technique, which has the advantages of being highly selective, applicable to a wide range of metal ions, requiring minimal sample handling, and permitting real-time analyses and speciation studies [8,9], was introduced to simulate metal transport through biological membranes [10]. PLM systems have been applied to the transport and separation of organic and inorganic chemical species. In the case of metal species, the organic solution contains an extractant that facilitates their transport through the membrane. The transport mechanism depends on both the chemical properties of the extractant, which can be complexing agents, solvating agents or ion exchangers and the chemical species to be transported [11,12,13]. Acidic cationic exchangers such as alkylphosphoric acids are highly versatile and have been applied to extract different metal ions such as Co(II), Ni(II), Zn(II), Fe(III), Cu(II), Ca(II), rare earth and V(V). The most widely used cationic exchanger is bis-(2-ethylhexyl)phosphoric acid (D2EHPA), whose main advantages are its chemical stability, good extraction kinetics, high load capacity, low solubility in the aqueous phase, and its commercial availability [14]. D2EHPA is a carrier that in membrane processes presents a great selectivity towards Zn(II) in comparison with other metals such as Cu(II), Ni(II), Cd(II), Ca(II), Mn(II), Fe(III), and Al(III), as has been demonstrated in previous membrane process studies [15,16]. In a particularly interesting study, a device based on a polymer inclusion membrane (PIM), in which D2EHPA was entrapped in a polyvinyl chloride matrix, was applied to determine the time-weighted average total Zn concentration in pond water [17]. Recently, the free Zn concentrations in a hydroponic media in the absence and presence of organic ligands was determined with a D2EHPA-based PIM device and the metal fluxes presented a good correlation with those obtained when Zn(II) is accumulated in potato plant roots [18]. The main advantage of PIM over PLM is its high stability, although the metal fluxes through the membrane are lower in the case of PIM [19]. 

Hollow fiber-supported liquid membranes (HFSLM) are based on the use of a microporous support fiber impregnated with an organic phase. The metal species are transported across this organic phase from an aqueous phase (donor phase) to another aqueous phase (stripping phase) inside the fiber. Through this simple system, which does not require expensive equipment, metal speciation studies can be performed and metal can be preconcentrated, allowing the achievement of high enrichment factors. This PLM configuration was proposed for the passive sampling of Pb(II) and for the evaluation of Cu(II), Ni(II) and Zn(II) bioavailabilities in complex aqueous media. In these studies, organic solutions of the complexing agent Kelex-100 were used in the liquid membrane phase [20,21,22,23]. A mixture of a Kryptofix 22DD and lauric acid as carriers in an HFSLM system was applied to evaluate Zn speciation in plant nutrient solutions [24]. In the development of an HFSLM passive sampler device for several divalent metals, including Cu(II) and Zn(II) in water samples, a mixture of a crown ether and D2EHPA dissolved in hexylbenzene was used [25].

The goal of the present study was to design an HFSLM system for Zn speciation analysis that will be able to allow selective free Zn(II) transport under different chemical conditions such as the presence of ligands and other metal ions in the donor solution. The feasibility of this method to estimate free Zn(II) concentrations in real water samples was also studied.

## 2. Materials and Methods

### 2.1. Reagents and Solutions

Aqueous solutions of Zn(II) were prepared by dilution of the corresponding stock solution prepared with ZnSO_4_·7H_2_O (Fluka, Steinheim, Germany). The ionic strength was adjusted with sodium nitrate (Panreac, Barcelona, Spain), and HNO_3_ and Ca(NO_3_)_2_ (Panreac, Barcelona, Spain) were used to prepare the stripping solutions. Calibration standards for the determination of Zn, Cu and Fe were prepared by dilution of the corresponding individual standard stock solutions (1000 mg L^−1^) for atomic spectroscopy (Sigma-Aldrich, St. Louis, MO, USA). Other reagents used were citric acid monohydrate (Merck, Darmstadt, Germany) and disodium ethylenediaminetetraacetate dihydrate (Panreac, Barcelona, Spain). Individual stock solutions of Cu(II), Fe(III), Ca(II) and Mg(II) were prepared from CuSO_4_·5H_2_O, Fe(NO_3_)_3_·9H_2_O Ca(NO_3_)_2_·4H_2_O and MgSO_4_·7H_2_O, respectively. All chemicals were of analytical reagent grade and the solutions were prepared with ultrapure water obtained by purification through a MilliQ Plus system (Millipore, Bedford, MA, USA). Bis-(2-ethylhexyl)phosphoric acid (D2EHPA) and dodecane (Sigma Aldrich, St. Louis, MO, USA) were used as received. Hydrophobic polypropylene fibers Accurel^®^ PP S6/2 (Membrana GmbH, Wuppertal, Germany) of 30 cm length were used as the liquid membrane support. These fibers had the following characteristics: Thickness: 0.45 mm, inner diameter: 1.8 mm, pore diameter: 0.2 µm and membrane porosity: 73%, which resulted in an effective inner membrane area of 5.65 cm^2^.

### 2.2. Instrumentation

Metal determination in both donor and stripping phases was done by atomic emission spectrometry with an ICP-AES instrument (Varian Liberty RL, Victoria, Australia) at 213.86 nm for Zn, 238.04 nm for Fe and 327.395 nm for Cu. The pH measurements of the aqueous samples were performed with a Crison Model GLP 22 pH meter (Barcelona, Spain).

### 2.3. Hollow Fiber Supported Liquid Membrane (HFSLM) Experiments

**Figure 1:**
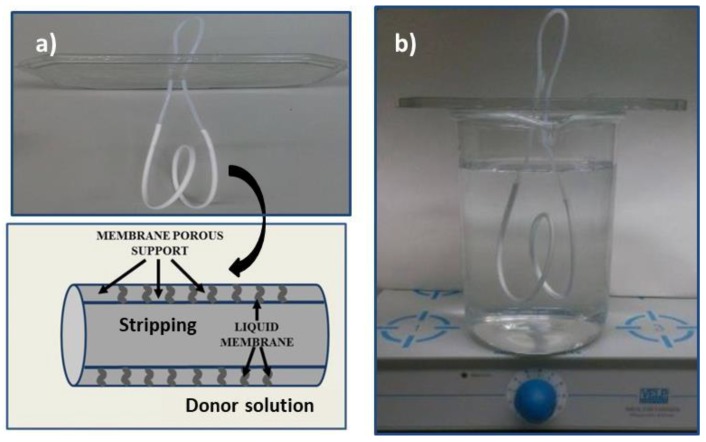
(**a**) Details of the hollow-fiber and scheme of the supported liquid membrane system; (**b**) the experimental set-up used in the HFSLM experiments.

The donor phase consisted of 500 mL of aqueous solution at different trace concentrations ranging from 0.1 to 2.5 mg L^−1^ with an ionic strength adjusted to 0.1 M with NaNO_3_. The liquid membrane consisted of an organic solution of D2EHPA in dodecane at different concentrations: 0.1, 0.25 and 0.5 M impregnated in the pores of a microporous tubular fiber (Figure 1a). The impregnation was carried out by soaking the 30 cm long fiber with the organic solution with the help of a syringe. Once impregnated, the excess of the organic phase was eliminated by immersing the fiber in ultrapure water. The stripping phase, composed of either 500 or 600 μL of aqueous acidic solutions, was introduced with a syringe into the lumen of the fiber once the impregnated fiber was immersed in 500 mL of the donor solution containing the metal ion. This solution was continuously agitated at 200 rpm (Figure 1b). When the experiment was completed, the stripping phase was aspirated with a syringe from the lumen of the fiber and was diluted with ultrapure water until 1 or 2 mL before being analyzed by ICP-OES. Two sets of six standard solutions were prepared to build the calibration curves for the determination of Zn. The first set ranged from 0.01 to 2.5 mg L^−1^ of Zn(II) in NaNO_3_ aqueous media and was employed for the metal determination in the donor solution. The second set, ranging from 20 to 250 mg L^−1^ of Zn(II), was prepared in the same media as the stripping solutions. When other metals such as Cu and Fe were present in the donor and stripping solutions, their concentrations were also determined by ICP-OES and the calibration standards were prepared following the same procedure as in the case of Zn. All experiments were carried out at ambient temperature (22 ± 1 °C).

Different trials were conducted by varying the composition of the stripping phase by using 0.1 M HNO_3_, 0.01 M HNO_3_ + 0.036 M Ca(NO_3_)_2_ and 0.0001 M HNO_3_ + 0.04 M Ca(NO_3_)_2_ solutions. The concentrations of the components were calculated in order to obtain a fixed ionic strength of 0.1 M in the stripping solution. 

Preliminary experiments were performed in order to establish the time required to achieve equilibrium in the HFSLM system using 500 mL of the donor solution containing 2 mg L^−1^ of Zn(II) in 0.1 M NaNO_3_ aqueous media, a 0.1 M D2EHPA in dodecane as the membrane phase, and 600 µL of 0.1 M HNO_3_ in the stripping phase. Different aliquots (2 mL) were collected from the donor aqueous phases at scheduled time intervals and the Zn concentration was determined by ICP-OES.

### 2.4. Chemical Speciation Software

The fractions of the different chemical species in the experimental aqueous media were calculated using the MEDUSA (Make Equilibrium Diagrams Using Sophisticated Algorithms) software [26] using the constants of the Hydra database that are included in the software. In order to compare the results of these calculations, Visual MINTEQ ver. 3.1 software [27] was also employed. 

### 2.5. Effect of Ligands on Zn Transport with the HFSLM System

To evaluate the influence of the presence of organic ligands in the donor solution on the transport of Zn(II) through the HFSLM system, two different complexing ligands, citric acid and disodium ethylenediaminetetraacetate (EDTA), were tested. The concentration of each ligand added to the donor solution was set considering both having the same initial Zn(II) in the aqueous media (~2 mg L^−1^), as in experiments performed without the presence of ligands, and having a measurable part of the Zn(II) not complexed by the ligands. Given this, Medusa software [26] was used to calculate the ligand concentrations to be added to the donor solution. Resulting donor solutions consisted of 500 mL of 1.5 mg L^−1^ citric acid and 2 mg L^−1^ of Zn(II) in a 0.1 M NaNO_3_ aqueous media (pH 5.5) for the citric ligand, and 500 mL of a 5 mg L^−1^ of EDTA and 2 mg L^−1^ of Zn(II) solution at pH 5.8 in 0.1 M NaNO_3_ aqueous media in the case of EDTA. In these experiments, the membrane phase was composed of 0.1 M of D2EHPA in dodecane and the stripping phase was 600 µL of a 0.1 M HNO_3_ solution. After 7 h, both the remaining Zn(II) concentration in the donor phase and the Zn(II) transported to the stripping phase were determined by ICP-OES.

### 2.6. Selectivity of the Membrane System

The selectivity of the membrane system towards Zn(II) was evaluated in the presence of other metals ions such as Cu(II) and Fe(III) in the donor solution. The first set of experiments was performed with a donor solution composed of 0.5 mg L^−1^ of Zn(II) and 0.5 mg L^−1^ of Cu(II) in 0.1 M NaNO_3_ aqueous media. In the second set of experiments, the donor solution consisted of equal concentrations (0.8 mg L^−1^) of Zn(II), Cu(II) and Fe(III) in 0.1 M NaNO_3_ aqueous media. To evaluate the selectivity of the membrane in presence of Ca(II) and Mg(II), which are present in natural waters, a donor solution containing equal concentrations (2 mg L^−1^) of Zn(II), Cu(II) and Fe(III), 120 mg L^−1^ of Ca(II) and 40 mg L^−1^ of Mg(II) in 0.1 M NaNO_3_ was used. In all the experiments, a 0.1 M D2EHPA/dodecane solution was used as the membrane phase and 500 µL of a 0.1 M HNO_3_ solution as the stripping phase. Experiments were performed for 7 h and both the initial concentration in the donor solution and the concentration in the stripping solution of each metal ion were determined by ICP-OES.

### 2.7. Performance of the HFSLM System in Natural Waters

To evaluate the applicability of the HFSLM device to determine Zn(II) concentration in natural water systems, different water samples were collected from a mine drainage stream and from the Osor River (Girona, Spain) in which particularly high levels of Zn have been found, ranging from 0.33 to 0.62 mg L^−1^, as a result of mining activity [28]. The water samples were characterized by determining their pH, conductivity, total organic carbon (TOC), total inorganic carbon and ionic composition. An ICP-OES semi-quantitative analysis was also performed to detect the presence of other metal ions. 500 mL of Osor River samples were used as the donor solution for the HFSLM experiments. In order to assess the effect of filtration on Zn transport efficiency, a set of experiments was also conducted after passing the water through a 0.45 µm cellulose acetate filter (Sigma Aldrich, Steinheim, Germany). 

## 3. Results and Discussion

The HFSLM system was designed to find both the best chemical and hydrodynamic conditions for the efficient transport of target metals. To this end, the selection of a carrier that is able to transport free Zn (II), such as bis-(2-ethylhexyl)phosphoric acid (D2EHPA), is essential.

The transport of Zn(II) through the membrane containing D2EPHA is explained by the following reactions [29]:

Extraction: Zn^2+^_(donor)_ + (3/2)(HL)_2(mem)_ ⇆ ZnL_2_.HL_(mem)_ +2 H^+^_(aq)_ (1)

Stripping: ZnL_2_·HL_(mem)_ + 2 H^+^_(donor)_ ⇆ (3/2) (HL)_2(mem)_ + Zn^2+^_(donor)_ (2)

where (HL)_2_ stands for the dimeric form of D2EHPA and subscripts donor and strip refer to the donor and stripping solutions and mem to the membrane phase. The driving force behind the transport is the difference in acidity between the two solutions separated by the liquid membrane.

### 3.1. Kinetics of the Zn(II) Transport through the HFSLM

**Figure 2:**
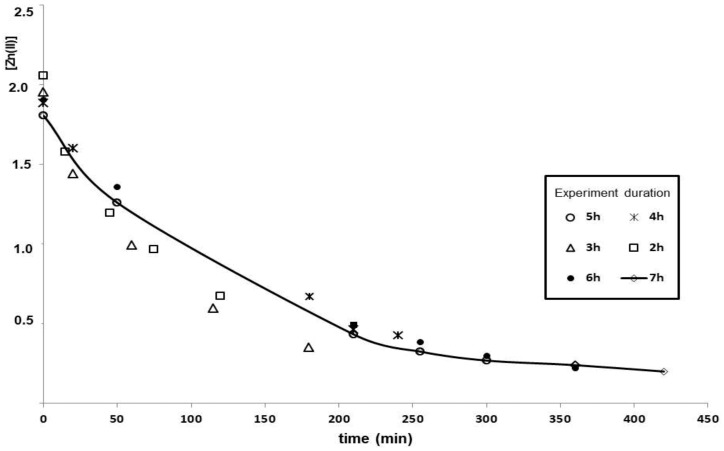
Kinetics of the Zn(II) extraction in the liquid membrane system. The Zn(II) concentration in the donor solution was monitored at prefixed periods of time in six experiments of different duration. Initial Zn(II) concentration: 2 mg L^−1^ in 0.1 M NaNO_3_ and membrane phase: 0.1 M bis-(2-ethylhexyl)phosphoric acid (D2EPHA) in dodecane. Stripping phase: 600 µL of 0.1 M HNO_3_.

As can be seen in Figure 2, equilibrium was achieved after 7 h under the tested conditions. The main part of the depletion in the Zn(II) concentration took place in the first 4 h (~82%) and ~10% in the final 2 h. Hence, the duration of later experiments was set at 7 h. 

### 3.2. Influence of D2EHPA Concentration on Zn Transport

The carrier concentration was studied at three levels: 0.1, 0.25 and 0.5 M of D2EPHA in dodecane. The results obtained were represented as the extraction efficiency of Zn(II) calculated by:

(3) $$\;E\%=\;\frac{{\left[\mathrm{Zn}\left(\mathrm{II}\right)\right]}_{\mathrm{donor}\left(0\right)}-{\left[\mathrm{Zn}\left(\mathrm{II}\right)\right]}_{\mathrm{donor}\left(t\right)}}{{\left[\mathrm{Zn}\left(\mathrm{II}\right)\right]}_{\mathrm{donor}\left(0\right)}\;}\;\%\;$$

where [Zn]_donor(0)_ is the initial Zn concentration in the water sample whereas [Zn]_donor(t)_ is the metal concentration in the source solution at the end of the experiment (7 h).

**Figure 3:**
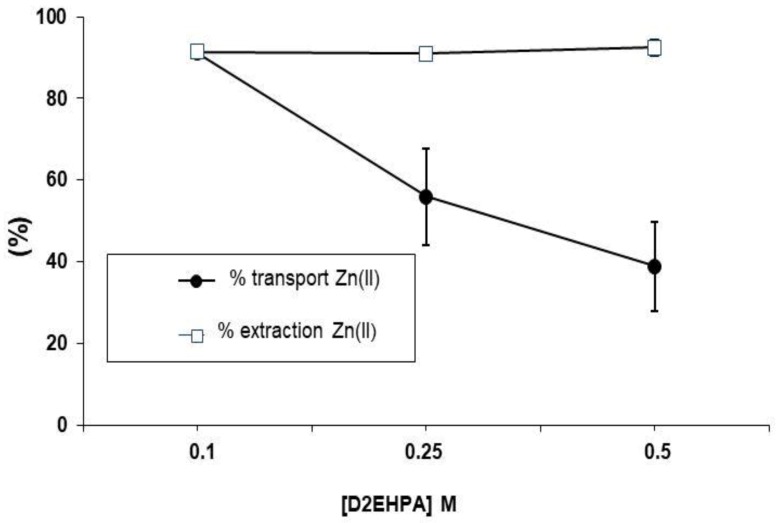
Effect of the D2EPHA concentration on the extraction and transport of Zn (*n* = 3). [Zn(II)] = 2 mg L^−1^ in 0.1 M NaNO_3_. Stripping phase: 600 µL of 0.1 M HNO_3_.

The percentage of Zn transported to the acceptor phase is calculated as:

(4) $$\;T\%=\;\frac{{\mathrm{mass}\;\mathrm{Zn}}_{\mathrm{strip}\left(t\right)}\;}{{\mathrm{mass}\;\mathrm{Zn}}_{\left(\mathrm{donor}\right)0}\;}\;\%\;$$

where the mass Zn_(strip)t_ is the mass of Zn transported to the stripping phase at the end of the experiment and mass Zn_(donor)0_ is the initial mass of Zn in the donor phase. Both mass values were calculated taking into account the measured Zn concentrations and the corresponding volumes. As can be seen in Figure 3, the Zn(II) extraction efficiency (>90%) was independent of the carrier concentration. However, Zn(II) transported to the stripping phase decreased when the D2EPHA concentration in the membrane phase was increased, indicating that the metal remained in the liquid membrane. This fact can be explained by the greater viscosity of organic solutions with 0.25 and 0.5 M of the extractant that hinders the diffusion of Zn-D2EPHA complexes through the liquid membrane to the stripping solution interface. Further experiments were performed with a carrier concentration of 0.1 M to ensure the transport of all the extracted Zn(II).

### 3.3. Influence of the Stripping Solution Composition on Zn Transport 

**Figure 4:**
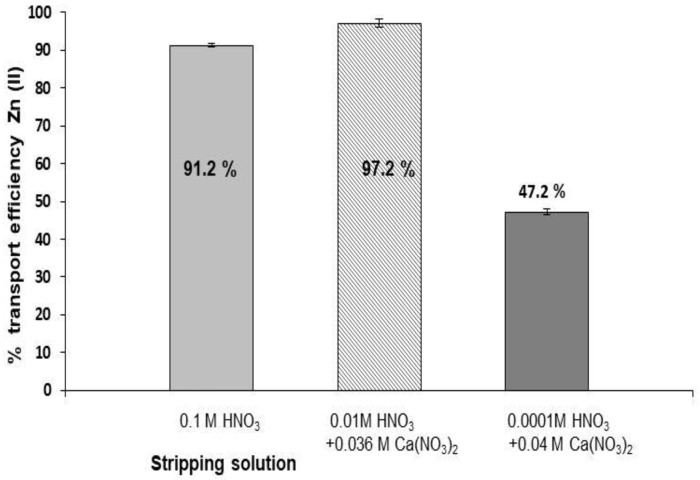
Effect of the composition of the stripping solution (*n* = 3). [Zn(II)] = 2 mg L^−1^ in 0.1 M NaNO_3_ and membrane phase: 0.1 M D2EPHA in dodecane. V_Strip_ = 600 µL.

The effect of the composition of the stripping phase was studied by conducting transport experiments with different compositions, such as a 0.1 M HNO_3_ solution and solutions containing both HNO_3_ and Ca(NO_3_)_2_ at different concentrations and maintaining the ionic strength at 0.1 M. The use of acidic solutions was intended to facilitate the cation exchange of the protons by Zn in the membrane-stripping phase interface, allowing the transport of the metal ion to the receiving solution (Equation (2)). Moreover, the presence of Ca^2+^ ions, which also form complexes with D2EPHA, is expected to enhance Zn(II) transport by exchanging this metal with Ca^2+^ at the same time as the cation exchange of Zn(II) by protons is taking place. As can be seen in Figure 4, transport efficiencies higher than 90% were obtained with a 0.1 M HNO_3_ solution and a stripping phase solution composed of 0.01 M HNO_3_ and 0.036 M Ca(NO_3_)_2_. The small differences in the transport efficiency values between both solutions together with the fact that in further experiments using 0.1 M HNO_3_ (Section 3.5), higher mean transport efficiencies (93.2%) were obtained, led us to consider that the presence of Ca(II) in the stripping solution did not have a significant effect on Zn transport. However, a decrease in the acidity of the solutions containing Ca(NO_3_)_2_ from 0.01 M to 0.0001 M HNO_3_ while the concentration of calcium is of the same order (0.04 M) resulted in a decrease in the percentage of Zn transport (47.1%), showing that the recovery of Zn(II) in the stripping solution is mainly due to the pH gradient through the membrane system. For this reason, the system was more efficient when the stripping solution had an acidic pH (1–2) and the donor solution had a pH of 5.5–6. Similar results were obtained when an initial Zn(II) concentration of 0.5 mg L^−1^ was used. In further experiments, a 0.1 M HNO_3_ solution was used as the stripping phase for simplicity.

### 3.4. Effect of the Presence of Zn Ligands on the HFSLM System

**Figure 5:**
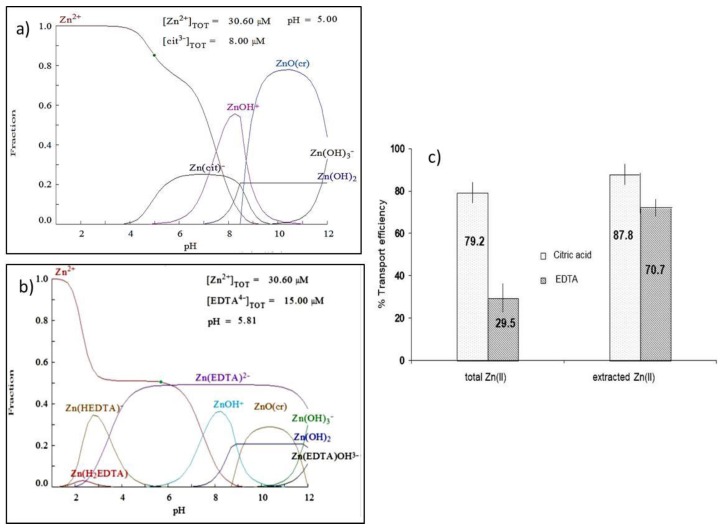
Effect of the presence of ligands on the Zn transport. (**a**) Species distribution of Zn in the presence of 8 µM (1.5 mg L^−1^) of citric acid; (**b**) species distribution of Zn in the presence of 15 µM (5 mg L^−1^) of EDTA; (**c**) transport efficiency of Zn (total and free) in the presence of 1.5 mg L^−1^ of citric acid or 5 mg L^−1^ of EDTA in the donor phase (*n* = 3). [Zn(II)]_tot_ = 2 mg L^−1^, membrane phase: 0.1 M D2EPHA in dodecane and stripping phase: 600 µL of 0.1 M HNO_3_.

In order to evaluate the effect of the presence of ligands in the efficiency of zinc transport, disodium EDTA salt and citric acid at different concentrations were added to the donor solutions. These two ligands were chosen since they have different complexation capacities towards Zn(II) and form negatively charged complexes with the metal that cannot be transported through the liquid membrane. In the case of EDTA, 49.6% of the total Zn(II) concentration is in the form of free Zn(II), according to the calculations made using Medusa software [26], whereas, for citrate, the free Zn(II) reaches a value of 78.9%. Both fraction diagrams are presented in Figure 5a,b. 

In Figure 5c, the Zn transport efficiencies are represented considering the initial and the extracted Zn(II) concentrations. The latter was calculated after determining the remaining Zn(II) concentration in the donor phase at the end of the experiment. As can be seen, the percentage of the total Zn(II) concentration transported in the presence of citric acid was 79.2%, which corresponds to the percentage of free Zn(II) concentration calculated by the program Medusa in the same chemical conditions (78.9%). However, in the experiments performed with EDTA, the Zn transport efficiency (29.5%) was lower than the free Zn(II) percentage calculated by the software (49.6%). These results point out that in the case of citric acid, all free Zn(II) was transported whereas, in the case of EDTA, only a 59.5% of the calculated free Zn(II) was transported. Zn extraction efficiencies, *E*%, in the presence of citric acid and EDTA in the donor solution were 87% and 38%, respectively. The comparison of the *E*% and *T*% values led us to confirm that the presence of citric acid did not affect both extraction and transport Zn (II) efficiencies and, on the contrary, both processes were affected when EDTA was present, even at small concentrations. Also in Figure 5c, transport efficiencies of the extracted Zn(II) in the donor solution, which was considered to be free Zn(II) ions, are represented. Transport efficiencies of 87.8% and 70.7% were obtained in the presence of citric acid and EDTA, respectively. These values are compared with the percentage of free Zn(II) transport (90%) under the same chemical conditions and without the presence of ligands resulting in a significantly lower value (70.7%) being obtained when EDTA was present in the donor solution. From these results, we can suggest that the presence of strong Zn(II) chelators such as EDTA in the aqueous solution affect the extraction and transport of free Zn(II) through the membrane.

### 3.5. Selectivity of the HFSLM System towards Different Metals

**Figure 6:**
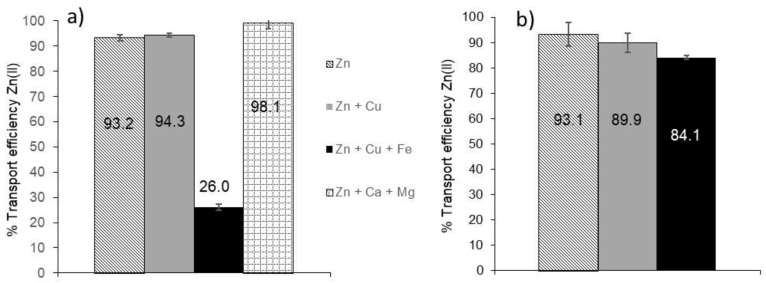
Transport efficiency of Zn(II) in presence of other metals (*n* = 3). (**a**) Initial concentrations of Zn(II), Cu(II) and Fe(III): 2 mg L^−1^; [Ca(II)] = 120 mg L^−1^ and [Mg(II)] = 40 mg L^−1^; (**b**) initial concentrations of Zn(II), Cu(II) and Fe(III): 0.8 mg L^−1^. In both trials: membrane phase: 0.1 M D2EPHA in dodecane and stripping phase: 600 µL of 0.1 M HNO_3_.

The selectivity of the liquid membrane system towards Zn(II) was evaluated in the presence of other metals ions such as Cu(II) and Fe(III) in the donor solution since it has been reported that D2EHPA is able to extract both metals [30,31,32]. The initial concentrations for all the metals were 0.5, 0.8 and 2 mg L^−1^. Other experiments were performed with a donor solution of 2 mg L^−1^ of Zn(II) and 120 mg L^−1^ of Ca(II) and 40 mg L^−1^ of Mg(II), which were the Ca and Mg concentrations determined in river water samples. As can be seen in Figure 6a,b, Zn(II) transport was not affected by the cations Cu(II), Ca(II) and Mg(II) present in the donor solution. However, Zn(II) transport was affected by the presence of Fe(III). When the donor solution contained 2 mg L^−1^ of Fe(III), Zn(II) transport decreased from 93% (when no Fe(III) was present) to 26% (Figure 5a) whereas at a lower Fe(III) content (0.8 mg L^−1^) Zn transport was 84% (Figure 6b). The analysis of the stripping phase revealed that iron was only transported in 6%. Therefore, it is believed that this metal is highly hydrolyzed at the relatively low acidity conditions (pH 5.5) employed in the donor solution [33], resulting in the formation of Fe(OH)_3_ that can precipitate in the donor solution affecting the diffusion of Zn(II) through the membrane phase. At a lower initial concentration of both metals (0.5 or 0.8 mg L^−1^), a much lower quantity of iron hydroxide was formed and, for that, the transport of Zn(II) was less affected (84%).

### 3.6. Preconcentration of Zinc

HFSLM configurations provided a large area that allowed separation and preconcentration to be performed in a single step with high enrichment factors. The enrichment factor (*EF*) reflects how many times the concentration of the metal in the stripping phase is increased compared to the initial sample concentration and it is defined as:

(5) $$\;EF=\;\frac{{\left[\mathrm{Zn}\left(\mathrm{II}\right)\right]}_{\mathrm{strip}\left(t\right)}}{{\left[\mathrm{Zn}\left(\mathrm{II}\right)\right]}_{\mathrm{donor}\left(0\right)}\;}\;$$

where [Zn]_strip(t)_ is the concentration of Zn in the stripping solution at 7 h and [Zn]_donor(0)_ is the metal concentration in the donor solution at the beginning of the extraction. 

Mean enrichment factors of 730 ± 50 for Zn(II) were obtained under the optimal conditions, which consisted of a 0.1 M D2EHPA solution in dodecane as the liquid membrane phase, 600 µL of 0.1 M HNO_3_ as the stripping phase and 500 mL of a Zn(II) solution (0.5, 0.8 or 2 mg L^−1^) in an 0.1 M NaNO_3_.

### 3.7. Performance of the HFSLM System in Natural Waters

**Figure 7:**
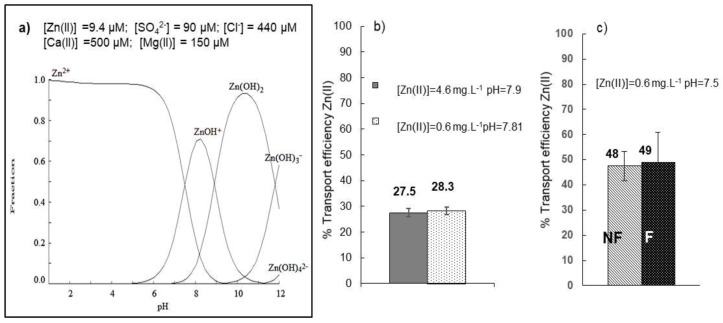
Performance of the HFSLM in natural waters. (**a**) Species distribution of Zn under the chemical conditions of Osor River water (conductivity: 130.3 µS cm^−1^; TOC: 3.53 mg C L^−1^; (**b**) percentage of Zn(II) transported in two different water samples (*n* = 3); (**c**) the effect of filtration in the percentage of Zn transport F: filtrated sample; NF: non-filtrated (*n* = 3). Membrane phase: 0.1 M D2EPHA in dodecane and stripping phase: 500 µL of 0.1 M HNO_3_.

Water samples were collected at two different points in the area of the Osor River (Girona, Spain) and were characterized. The total Zn(II) concentration in the Osor River was found to be 0.6 mg L^−1^ whereas a higher concentration (4.6 mg L^−1^ ) was determined in a stream (mine drainage) that was created after rainfall leading from the closed mine to the river. The total Fe concentrations in both samples were 0.01 and 0.1 mg L^−1^, respectively, while the total Cu concentration was below the detection limit with a pH of 7.8–7.9. The developed HFSLM system was applied to evaluate the free concentration of Zn(II) in these samples. Despite the fact that the HFSLM system was tested using ultrapure water at pH 5.5 and the real water samples had a higher pH, this was not adjusted in order to not disturb the chemical speciation of the water sample. The pH of the sample is a very important parameter as it strongly affects the hydrolysis of Zn(II). As can be in Figure 7a, the main species present under the chemical conditions of the River Osor water samples was Zn(OH)^+^, which had a percentage of ~65%, followed by 26% of Zn(II) and 9% of soluble Zn(OH)_2_. The percentage of Zn(II) transported through the membrane system in the two water samples was very similar with mean values of 27.5 ± 1.6% and 28.3 ± 1.5%, which were independent of the initial Zn(II) concentration. These experimental results are in agreement with the calculated fraction of free Zn(II) (26%), showing that only free Zn(II) ions were transported through the HFSLM system. It is important to remark that Medusa software do not include information about the formation of organic metal complexes with dissolved organic matter and that in a recent publication a decrease in the transport efficiency of Ni(II) in a PLM system was observed when the humic acid concentration increased [34], showing that the presence of dissolved organic matter affects metal transport through the liquid membrane.

In order to study the effect of the presence of particulate matter on the performance of the HFSLM system, a third water sample was taken from the Osor River and transport experiments were performed with filtered and non-filtered water. This water sample was also characterized and presented a similar ionic composition as the previously collected samples with a total Zn(II) concentration of 0.6 mg L^−1^ and a pH = 7.5. This pH value did not vary after water filtration. No significative differences were observed in the Zn transport efficiencies between the filtered and non-filtered samples (Figure 7c). This result can be explained by the relatively low TOC (3.53 mg L^−1^) of this sample. As can be seen in Figure 7c, the Zn transport efficiency in this water sample was higher (44 ± 2.3%) than in the other samples collected in the same river (Figure 7b) as a result of the presence of a higher percentage of free Zn(II) ions (48%). The fraction diagram (Figure 7a) illustrates the variations of the different Zn species as a function of pH. This diagram was calculated with Medusa software under the chemical conditions of the water samples. In all the experiments performed with real water samples, the percentage of free Zn transported was similar to the calculated free Zn fraction under the same chemical conditions, confirming the selective transport of free Zn (II) species. 

## 4. Conclusions

In this study, a simple pH-driven HFSLM system was developed and successfully applied to estimate free Zn(II) concentrations in river water samples. A quantitatively free Zn(II) transport (pH 5.5) was obtained under the optimized conditions, which consisted of a 0.1 M D2EHPA solution in dodecane as the liquid membrane phase and 600 µL of 0.1 M HNO_3_ as the stripping phase. The effect of the presence of organic ligands on the free Zn transport was evaluated by adding citric acid and EDTA, which form negatively charged complexes with Zn(II), to the donor phase. The efficiency of free Zn transport was only affected in the case of EDTA, resulting in 70.7% of the free Zn fraction being recovered in comparison with the 87.8% recovery that was achieved when citric acid was added. The selectivity of the membrane system towards Zn(II) was high as only this metal ion was transported in the presence of equal concentrations of Cu(II) and Fe(III) in the donor solutions and of Ca(II) and Mg(II) at the same concentration levels as are found in natural waters. The feasibility of the HFLSM system to estimate free Zn(II) concentrations in river water samples, which contained different total Zn(II) concentrations at different pHs has been demonstrated by analyzing three real water samples. The results obtained agree with the calculated free Zn fraction under the same chemical conditions, confirming that a quantitative transport of free Zn takes place. The HFSLM system developed here allows the preconcentration and transport of free Zn(II) in natural water samples, can be applied to study Zn speciation in environmental studies as well as to estimate trace concentration levels of free Zn(II) in complex aqueous matrices.
